# Susceptibility of Environmentally Friendly Sheep Wool Insulation Panels to the Common Clothes Moth *Tineola bisselliella* in Laboratory Assays

**DOI:** 10.3390/insects10110379

**Published:** 2019-10-31

**Authors:** Luca Ruiu, Ignazio Floris

**Affiliations:** Dipartimento di Agraria, Università degli Studi di Sassari, 07100 Sassari, Italy; ifloris@uniss.it

**Keywords:** Tineidae, pest management, sheep, borate, insulation

## Abstract

In this study the resistance opposed to *Tineola bisselliella* larvae by a commercial sheep-wool panel incorporating borate salts was determined under laboratory conditions. The susceptibility of clothes moth larvae to different concentrations of disodium octaborate tetrahydrate (DOT) incorporated in pure wool was also determined. The commercial wool panel showed a remarkable resistance to moth attack compared with pure untreated wool, and the damage to panel samples was limited to their surface. As a result of bioassays exposing larvae to pure wool treated with DOT, a concentration dependent effect was observed, achieving a good efficacy at an application rate between 40–100 mg/mL. This study highlights the need to protect wool-based construction material with appropriate insecticidal (antifeedant or repellent) substances and supports the development of eco-sustainable solutions.

## 1. Introduction

The use of renewable materials for building insulation has been characterized by a significant increase in recent years, as a result of specific incentive systems and a generally increased awareness of economic and ecology related advantages [1]. Among the variety of newly proposed solutions for building wall insulation, the case of wool panels obtained by different technological processes from locally produced sheep wool is significant [2]. Despite the clear advantages associated with the use of such materials, their biodegradability can make them more susceptible to adverse environmental factors, among which a main role is played by infestations of the common clothes moth *Tineola bisselliella* (Hummel) (Lepidoptera: Tineidae). Larvae of this cosmopolitan pest feed on keratin-rich sources of food, exploiting an unusual ability to digest this protein [3], and cause economically significant damages to different items, including clothes, furnishings, books, and art works [4]. Conventional methods of control are based on the use of insecticides, especially synthetic pyrethroids (i.e., permethrin) applied during the manufacturing process, and exceptionally on the use of fumigants [5]. Because of the safety and health concerns associated with the use of chemicals, a more eco-sustainable approach, including accurate adult detection and monitoring by pheromone traps is recommended [6]. When applicable, control measures with lower environmental impact involve the use of physical barriers to protect susceptible items, brushing or vacuum-cleaning, and treatment at extreme temperatures [4]. Finally, the employment of natural enemies like hymenopteran parasitoids have been proposed, but they have not found an actual commercial use [7].

Protection of sheep wool panels against clothes moth is often based on the use of approved insecticidal (chemical) products. However, to avoid the employment of synthetic chemicals, in line with an eco-sustainable approach, specific physical treatments can be applied to the product during manufacturing procedures. These may involve heating phases by which possible eggs and juvenile stages are killed by high temperatures [8]. Finished products must then be preserved up to commercialization and installation into buildings, normally inside wall cavities. Additional treatments of panels with common construction materials may indirectly confer resistance against clothes moths. For instance, the incorporation of borate salts in such materials as a fire retardant is expected to produce antiseptic and insecticidal supplementary effects [9,10]. While the insecticidal properties of boron-containing compounds are known, their potential in protecting new construction materials like wool panels, as an alternative to synthetic chemicals (i.e., pyrethroids), deserves specific investigation.

The main objectives of this study were (1) to evaluate the resistance opposed to larvae by a commercial sheep-wool panel treated with borate salts and (2) to determine the susceptibility of clothes moth larvae to disodium octaborate tetrahydrate (DOT) incorporated in pure wool.

## 2. Materials and Methods

### 2.1. Materials and Insecticidal Preparations

Disodium octaborate tetrahydrate (DOT) technical powder solutions used in bioassays were fresh prepared from technical powder (CAS N. 12280-03-4, Società Chimica Larderello, Milan, Italy) by water mixture just before use.

Commercial samples of sheep wool panels TECNOLANA were provided by the manufacturer (Brebey Scarl, Cagliari, Italy). Pure fine wool used in bioassays was obtained from Sardinian sheep, a breed of domestic sheep from the island Sardinia (Italy), specialized for milk production, developed by crossing local lowland sheep, Merinos, and North African sheep. Before use, wool was washed with warm water and left to dry.

Eggs of *T. bisselliella* were provided by Istituto per lo Studio degli Ecosistemi, National Research Council (Sardinia, Italy). Insects were maintained on pure wool in a growth chamber at 25 °C and 70% R.H.

### 2.2. Resistance Bioassays

Resistance bioassays methods were based on International Organization for Standardization methods [11] with some adaptations. Briefly, groups of 10 young *T. bisselliella* larvae (2–3 mm long) were maintained in Petri dishes (3.5 cm diameter) containing pure wool or commercial wool panel samples in a growth chamber at 25 °C and 70% R.H. The weight of these wool samples was recorded before and after exposure to larvae, after eliminating larval excrements and other residues. As a control, pure wool and panel samples were maintained under the same conditions inside Petri dishes, but with no contacts with larvae, to measure their drop in physiological weight. Data were assessed and compared after 15 days, determining the average mass loss due to larval feeding activity. Damage to the wool sample was also estimated by attributing it a class of damage (degree: 1, no attack; 2, slight attack; 3, moderate attack; 4, strong attack. A, no holes; B, partially damaged fibers; C, small and sparse holes; D, large and deep holes) (Table 1). Larval mortality during bioassay was recorded daily. This experiment involved four replicates.

### 2.3. Concentration-Response Bioassays

Second instar *T. bisselliella* larvae were exposed to pure wool treated with different concentrations of disodium octaborate tetrahydrate. Pure wool samples were immersed for a few seconds in a water solution containing variable concentrations of DOT and left to dry before being used in bioassays. A range of concentrations (3, 6, 12, 24, 50, 100 mg/mL) was assayed in comparison with untreated control. The experimental design involved four replicated groups of 10 larvae maintained in Petri dishes (3.5 cm diameter) for each concentration assayed and for the control. Larvae were maintained at 25 °C and provided ad libitum with DOT treated wool. Insects were inspected daily to assess larval mortality. The whole experiment was repeated three times with different batches of larvae.

### 2.4. Statistical Analyses

Statistical analyses were performed with SAS software (version 9.1) with significance level set at α = 0.05 [12].

In experiments with the commercial wool panel, weight loss data were analysed using t-tests to compare the commercial wool panel with pure wool samples.

Repeated measures ANOVA (PROC MIXED) was used for overtime mortality data analysis, and means were separated using LSMEANS comparison (adjust = Tukey).

Linear regression analyses were used for analyzing the relationship between DOT concentration and larval mortality.

## 3. Results

### 3.1. Resistance Bioassays

**Figure 1:**
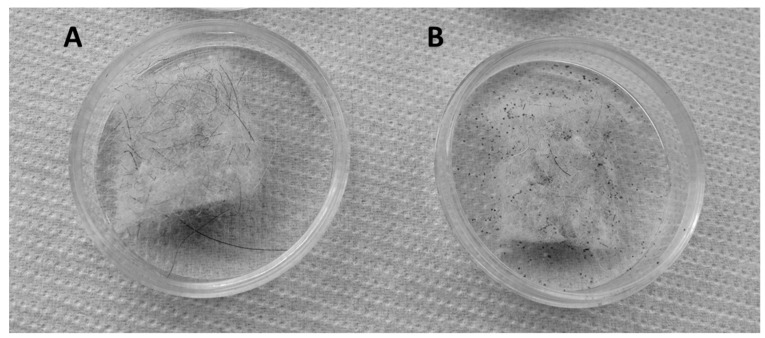
Samples of commercial wool panel (**A**) intact and (**B**) damaged on the surface by the action of clothes moth larvae.

The commercial wool panel showed a significant resistance to the attack of clothes moth larvae, compared with pure untreated wool. The damage to panel samples was limited to its surface, and the attributed classes of damage after 15 days contact with larvae ranged between 2B and 3C, corresponding to slight to moderate damage (Figure 1; Table 1).

**Figure 2:**
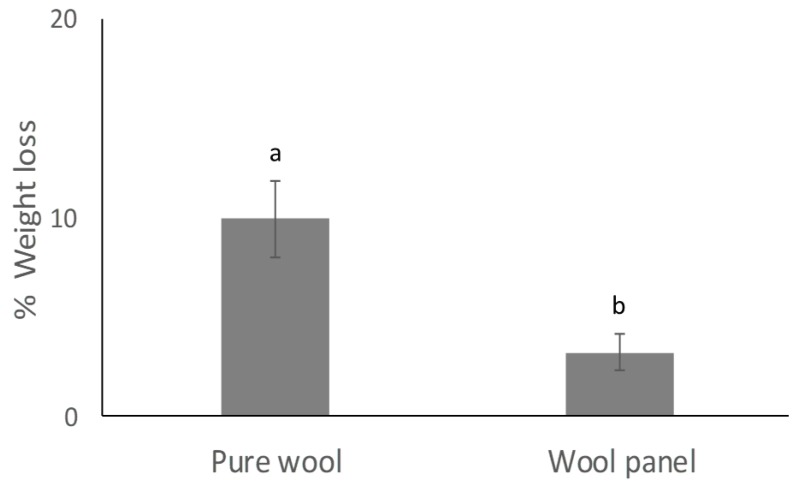
Weight loss of wool panel compared with pure wool samples exposed to clothes moth larvae for 15 days. Different letters above bars indicate significantly different means (*t*-test, *p* < 0.001).

Although exposure to larvae resulted in a weight loss of the wool panel, this was significantly lower (*t* = 10.50066; *p* = 0.00018) than that observed on pure wool (Figure 2).

**Figure 3:**
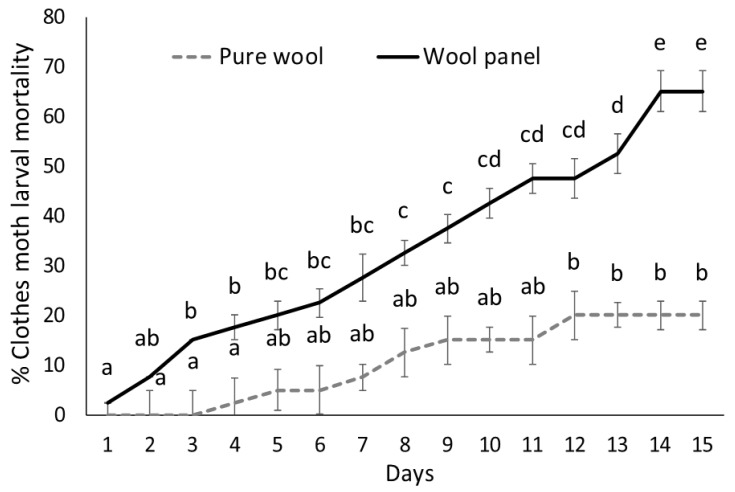
Over time mortality (mean percentage ± SE) of clothes moth larvae exposed to the commercial wool panel compared with pure wool. Different letters indicate significantly different means (ANOVA Mixed Proc., Tukey adjusted *p* < 0.05).

Larvae exposed to the commercial wool panel during the bioassay appeared to be less vital and their over-time mortality was significantly higher (F_1,6_ = 52.61, *p* = 0.0003), compared with larvae maintained on pure untreated wool (Figure 3). A significant mortality effect of exposure time was also detected (F_14,84_ = 44.41, *p* < 0.0001).

### 3.2. Concentration-Response Bioassays

**Figure 4:**
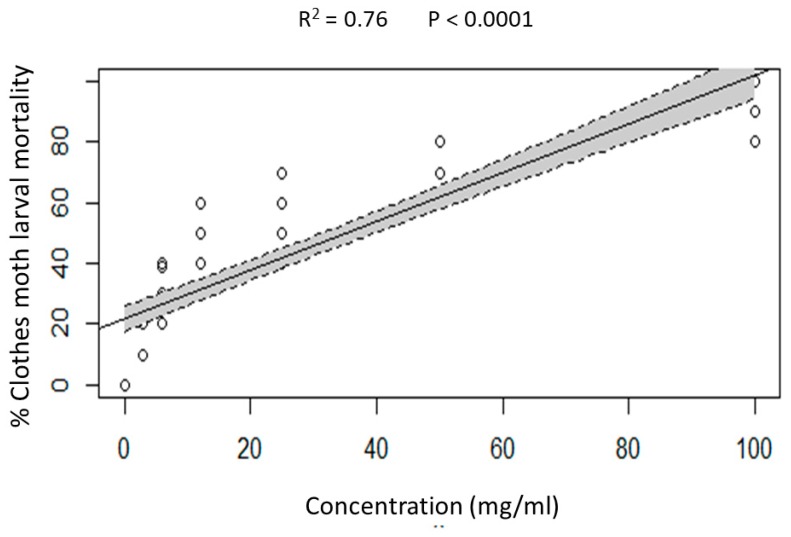
Linear regression plots with 95% confidence intervals (shaded areas) showing the predicted relationship between disodium octaborate tetrahydrate (DOT) concentration and mortality of clothes moth larvae exposed to treated wool for seven days.

Clothes moth larvae appeared to be significantly susceptible to disodium octaborate tetrahydrate (DOT) incorporated in pure wool, and the effect was concentration dependent (Figure 4). According to the result of linear regression analysis, larval mortality was shown to be positively correlated with the concentration of DOT in pure wool (adjusted *R*^2^ = 0.7627, F = 267.7, *p* < 0.0001).

## 4. Discussion

Wool panels represent a modern item exploiting the well-known insulation properties of sheep wool [13]. Replacing conventional materials with this renewable alternative is consistent with the principles of circular economy and ecology [1,2,14]. Despite its clear advantages, such material may be significantly affected by the action of environmental factors compromising its integrity. One of the risks associated with the use of wool is the possible degradation action by keratin-digesting insects, like larvae of *T. bisselliella* [3,15].

Based on the present study, clothing moth larvae appeared to be particularly voracious feeding on pure wool, while the commercial wool panels showed a significant resistance, even though they were not exempt from larval attack. Such resistance is likely to be related to the panel structure that incorporates other materials in addition to wool (i.e., glue, wood) and to an industrial manufacturing process conferring specific physical characteristics to the final product [16]. This may result in a reduced susceptibility to larval feeding activity, as we observed in bioassays in which the panel was exposed for 15 days to young larvae. On the other hand, the presence of different materials in the panel may provide food or refuge to other pests [15]. Part of the resistance properties against pests, might directly relate to the presence of substances absorbed or retained by the panel during manufacturing, such as borate salts. The significantly reduced viability we observed on clothes moth larvae developing on panel samples compared to pure wool supports a direct insecticidal action, which may explain the reduced feeding action on the panel.

The larvicidal properties of disodium octaborate tetrahydrate (DOT) were confirmed by the bioassays conducted in the present work, highlighting a concentration dependent effect [17]. The lethal concentrations observed in wool samples treated by immersion in a DOT solution in the range 40–100 mg/mL are consistent with the recommended doses of several borate salt-based commercial products normally applied to wood for protection against termites [18]. Boric acid and borate acts against insects by interfering with cell metabolism, blocking energy production mechanisms involving adenosine triphosphate [19]. In experiments conducted with boron-based biocides on wood against termites, maximization of the insecticidal action appeared to be directly related to the boron concentration in the wood, suggesting a key role of the formulation features on which depends the title of free boron irons in the substrate after application [20]. Studies with DOT and different termite species determined median lethal doses (LD50) ranging between 256.2 and 408.2 µg/g depending on the formulation used [21]. While such information is available for wood, more specific work on the interaction between available boron formulations and woolen manufactures is needed.

In order to achieve an increased protection against pests, formulation improvements should take into account several features of the target substrate [22]. In the case of wool panels, boron behaviour after application could be affected by the chemical-physical properties of both wool and additional materials that make up the structure of the panels [16]. Successful applications to panels could also be achieved by employing appropriate additives enhancing boron insecticidal potential or preserving its persistence in the treated substrate [23]. The lethal and antifeedant effects on clothes moth larvae we observed on wool panels treated with DOT during the manufacturing process support a good resistance of such substrate to this pest, compared to pure wool. However, for the purpose of an industrial standardization of the finished product, it is advisable to provide an ad hoc application of biocides that guarantee better efficacy and adequate persistence over time.

As for any biocidal application, safety issues are also to be considered. Boron is a naturally occurring material in the environment, and while it can be toxic at higher concentration, it is generally considered safe under certain levels in the terrestrial and aquatic environment [24]. For instance, the predicted no effect concentration (PNEC) values in the aquatic environment were shown to range around 1 mg boron/L [25]. Its appropriate use as a construction material preservative conferring specific resistance to pests, molds or fire should therefore be regarded as safe. Insecticidal products more frequently employed to protect wool from *T. bisselliella* are mostly based on conventional chemicals (i.e., pyrethroids) [4]. While these products are expected to achieve a good efficacy, the risks associated with their use, like environment contamination, side-effects on non-target organisms and the development of insect resistance, support the search for alternatives.

The availability of other active substances effective against pests threatening integrity of eco-sustainable construction materials, such as the clothes moth affecting wool panels, should stimulate more studies to develop persistent and biodegradable biocidal products. These may involve new solutions from the fast-growing industrial sectors of the biobased products deriving from plants [26,27] and microbials [28], or from nanotechnologies [29,30]. Such an approach may rapidly lead to wool-based construction materials with good resistance to pests and a further reduced environmental impact.

## 5. Conclusions

This study investigated the susceptibility of clothes moth larvae to borate salts incorporated in wool, determining the application dose range recommended to achieve satisfactory effects. Boron treatments on commercial sheep wool panels were able to confer a remarkable resistance against moth attack compared with pure untreated wool, limiting damage to the panel surface.

On the other hand, this study highlighted the need to develop new substances and industrially scalable methods to protect wool-based construction material, according with an effective and eco-sustainable approach.

## Figures and Tables

**Table 1:** Classes of damage attributed to wool samples exposed to clothes moth larvae.

Grade	Superficial Attack	Grade	Deep Attack
1	no attack	A	no holes
2	slight attack	B	partially damaged fibers
3	moderate attack	C	small and sparse holes
4	strong attack	D	large and deep holes
